# Induction of cancer testis antigen expression in circulating acute myeloid leukemia blasts following hypomethylating agent monotherapy

**DOI:** 10.18632/oncotarget.7326

**Published:** 2016-02-11

**Authors:** Pragya Srivastava, Benjamin E. Paluch, Junko Matsuzaki, Smitha R. James, Golda Collamat-Lai, Nadja Blagitko-Dorfs, Laurie Ann Ford, Rafeh Naqash, Michael Lübbert, Adam R. Karpf, Michael J. Nemeth, Elizabeth A. Griffiths

**Affiliations:** ^1^ Department of Medicine, Roswell Park Cancer Institute, Buffalo, NY, USA; ^2^ Department of Pharmacology and Therapeutics, Roswell Park Cancer Institute, Buffalo, NY, USA; ^3^ Center for Immunotherapy, Roswell Park Cancer Institute, Buffalo, NY, USA; ^4^ Department of Medicine, Division of Hematology/Oncology, University of Freiburg, Medical Center, Freiburg, Germany; ^5^ Eppley Institute for Cancer Research, Fred and Pamela Buffet Cancer Center, University of Nebraska Medical Center, Nebraska Medical Center, Omaha, NE, USA; ^6^ Department of Immunology, Roswell Park Cancer Institute, Buffalo, NY, USA

**Keywords:** acute myeloid leukemia, cancer testis antigen, NY-ESO-1, decitabine, immunotherapy

## Abstract

Cancer testis antigens (CTAs) are promising cancer associated antigens in solid tumors, but in acute myeloid leukemia, dense promoter methylation silences their expression. Leukemia cell lines exposed to HMAs induce expression of CTAs. We hypothesized that AML patients treated with standard of care decitabine (20mg/m2 per day for 10 days) would demonstrate induced expression of CTAs. Peripheral blood blasts serially isolated from AML patients treated with decitabine were evaluated for CTA gene expression and demethylation. Induction of *NY-ESO-1* and *MAGEA3/A6*, were observed following decitabine. Re-expression of *NY-ESO-1* and *MAGEA3/A6* was associated with both promoter specific and global (*LINE-1*) hypomethylation. *NY-ESO-1* and *MAGEA3/A6* mRNA levels were increased irrespective of clinical response, suggesting that these antigens might be applicable even in patients who are not responsive to HMA therapy. Circulating blasts harvested after decitabine demonstrate induced *NY-ESO-1* expression sufficient to activate NY-ESO-1 specific CD8+ T-cells. Induction of CTA expression sufficient for recognition by T-cells occurs in AML patients receiving decitabine. Vaccination against NY-ESO-1 in this patient population is feasible.

## INTRODUCTION

Acute myeloid leukemia (AML) is a heterogeneous disease with a broad spectrum of clinical presentations and heterogeneous response to therapy [1]. According to a 2014 estimate, more than half the patients diagnosed with AML in the United States will die from the disease [2]. A majority of AML cases are diagnosed in patients who are 65 years of age or older. The current standard of care for such patients is either conventional induction with cytarabine and an anthracycline, or treatment with a hypomethylating agent (HMA) such as decitabine or azacytidine [3, 4]. Either approach results in similar overall survival benefits and high rates of relapse. A potentially curative approach is allogeneic hematopoietic stem cell transplantation (aHSCT), which results in sustained remission in about half of eligible patients due to a graft-*versus*-leukemia effect [5]. Unfortunately, aHSCT is limited to fit patients with an available donor and is associated with significant treatment-related morbidity and mortality[6]. Nevertheless, the efficacy of aHSCT demonstrates the potential of the adaptive immune system to eradicate residual AML and provides a rationale for the development of alternative immunotherapeutic strategies [7].

The identification of appropriate antigens for T-cell directed immunotherapy in myeloid malignancy remains a challenge [8, 9]. CD123 and CD33 have shown some potential, but due to their co-expression on healthy hematopoietic cells, toxicity remains a significant barrier. Thus, identifying leukemia specific antigens would be a considerable advance towards solving this problem. Cancer testis antigens (CTAs) are a family of more than 200 X-linked and autosomal genes that are normally expressed in the embryonic ovary and the adult testis [10]. In all other adult tissues, expression of CTA family genes is limited due to epigenetic silencing of their regulatory elements. Aberrant expression of CTAs due to demethylation of their promoters has been found in multiple solid tumor types. [11–14]. Early and advanced clinical trials based on vaccination against CTAs have shown clinical benefit in lung, melanoma and ovarian cancer [15–17].

The utility of CTAs as viable tumor antigens in myeloid malignancies has been less studied. In contrast to solid tumors, hypermethylation of CTA gene promoters, in association with gene silencing is observed in leukemia cell lines and in primary specimens, limiting their potential [18–21]. We and others have demonstrated that treatment of AML cell lines, both *in vitro* and as tumor xenografts, with HMAs induces expression of CTAs [18–21]. In contrast with solid tumors, where HMAs have demonstrated limited clinical activity, these drugs are in routine use for the management of patients with AML [3, 4]. Induced expression of CTAs following HMA therapy would offer an opportunity for immunotherapy towards cells that re-express this antigen.

In this report, we evaluated the induced expression of CTA family members in peripheral blood samples serially isolated from AML patients with active disease undergoing decitabine monotherapy. We observed significant upregulation of both *New York-Esophageal Cancer-1* (*NY-ESO-1*) and *Melanoma Antigen Family A3/6* (*MAGEA3/A6*), established immunogenic tumor antigens. Expression of *NY-ESO-1* and *MAGEA3/A6* was associated with hypomethylation of their promoter regions. *NY-ESO-1* mRNA levels were increased in samples from patients who did not respond clinically to HMA therapy, suggesting that immunotherapies that recognize CTAs have the potential to benefit this population of patients for whom current therapies are limited. The induction of *NY-ESO-1* expression by decitabine resulted in the presentation of antigen at sufficient levels for recognition by NY-ESO-1 specific CD8+ T-cells. Together, our data indicate that immunotherapeutic approaches directed against CTAs are feasible within the clinical context of patients receiving HMAs for myeloid malignancy.

## RESULTS

### HMAs induce CTA expression in AML patients

**Figure 1:**
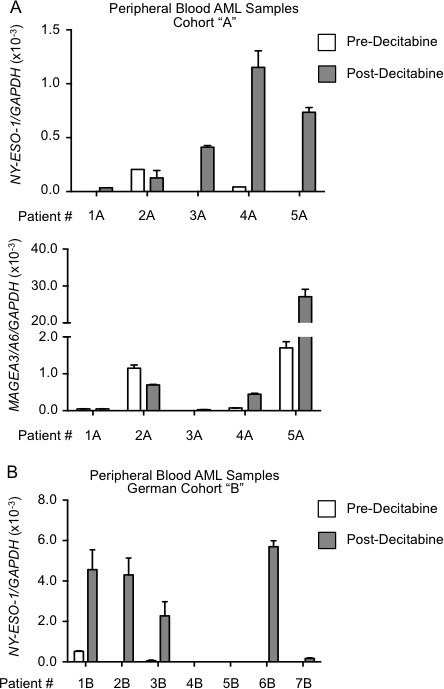
Induction of *NY-ESO-1* and *MAGEA3/A6* in AML peripheral blood cells following decitabine monotherapy Peripheral blood samples from Patient Cohort “A” (Roswell Park, *n* = 5) and German Cohort “B” (University of Freiberg, *n* = 7) (see Table 1 for clinical characteristics) were harvested pre-decitabine and post-decitabine. **A.** Quantitative PCR analysis of *NY-ESO-1* (top) and *MAGEA3/A6* (bottom) mRNA levels obtained pre-decitabine (white bar) and post-decitabine (gray bar) from Cohort “A”. mRNA levels were determined using absolute quantification and normalized to *GAPDH* mRNA levels (see Methods). Error bars depict SEM of 2 technical replicates for each patient sample. **B.** Quantitative PCR analysis of *NY-ESO-1* mRNA levels obtained pre-decitabine (white bar) and post-decitabine (gray bar) from German Cohort “B”.

To determine whether decitabine monotherapy resulted in CTA gene expression, we isolated RNA from serial peripheral blood samples harvested from AML patients during a first cycle of therapy. We began by examining expression of a panel of eight different CTA genes in two patients, one who had received decitabine 20mg/m^2^/day for 10 days and the other azacitidine at a dose of 75mg/m^2^/day for 7 days (Supplemental Figure 1A) [19, 20, 22, 23]. In these first two patients we observed low level mRNA induction of *MAGE* family members as well as *NY-ESO-1* and *X antigen family member 1* (*XAGE1*), but limited induction of *PAS domain containing 1* (*PASD1*)*, Preferentially Expressed Antigen in Melanoma* (*PRAME*) and *Sperm Autoantigenic Protein-17* (*SP17*). We went on to examine expression of *MAGEA1, MAGEA3/A6, NY-ESO-1* and *XAGE1* in a larger panel of five AML patients treated with decitabine 20mg/m2/day for 5-10 days as a single agent, clinical characteristics are presented in Table 1 (Cohort A). These patients demonstrated limited induction of *MAGEA1* (1/5 patients), but 3/5 patients showed induction of *XAGE1* (Supplemental Figure 1B). Induced mRNA expression of *NY-ESO-1* was seen in 5/5 patients. In concordance with the observed induction of gene expression, hypomethylation of the *NY-ESO-1* promoter was observed (Supplemental Figure 2). Induction of *MAGEA3/A6* was observed in 3/5 patients from this cohort (Figure 1A).

**Table 1:** Patient characteristics: cohorts “A” and “C”

Patient ID	Age	Sex	Karyotype	Response To DAC	Blast count (% of WBM)	Prior AML Rx
1A	54	F	normal	RD	90	Yes
2A	80	M	complex with del(5)der(7)	Early Death	29	No
3A	91	M	+8,del(12)(p12p13) x 2,+12,+21	Early Death	71	No
4A	73	M	t(11;17)(q13;p13)	RD	33.6	No
5A	65	M	del(7)(q22q36)	Early Death	26.4	No
**Patient ID**	**Age**	**Sex**	**Karyotype**	**Response To DAC**	**Blast count (% of WBM)**	**Prior AML Rx**
1C	84	F	t(3;3)(q21;q26),and idem,i(16)(p10)and idem,+12	RD	46	No
2C	81	M	t(8;19)(q24;q13.1) and t(1;16)(q12;q24),del(20)(q22.1q13.3)	CRp	77	Yes
3C	76	M	normal	CRp	52.8	No
4C	73	M	normal	RD	87.8	No
5C	80	M	del(20)(q11.2q13.3) and der(2)t(2;11)(q32;q13),del(20)(q11.2q13.3)	RD	30	No
6C	78	F	5q-, +8, +q22 and +q23.	RD	82	Yes
7C	68	M	complex with del(5), +8	CRi	28	No
11C	76	F	complex with -5, +8, t(5,12), t(16,17)	RD	71	No
12C	72	M	complex with del(9), del(22)	Early Death	22	No
13C	79	M	normal	CR	35	No
14C	70	M	+13	RD	98	No
15C	77	F	normal	CR	71.6	No
17C	75	F	MLL+; der(1)?t(1;1)(p36.1;q21),+4,t(9;11)(p22;q23)	RD	88	No
19C	75	F	complex with -5,-7,del(12),del(13)	RD	23	No
20C	86	M	+mar	RD	88	No
21C	46	F	complex	Early Death	ND	No
22C	64	M	normal	RD	90.8	Yes
23C	77	M	not done	Early Death	ND	No
24C	75	M	+8	CRp	ND	No
25C	80	F	not done	RD	57	No
26C	71	M	normal	HI-P Major	20	Yes
27C	77	F	normal	RD	41.5	Yes

Patient samples from cohorts A and C are marked an ID number followed by their respective letter. All patients were treated with decitabine 20mg/m2/d for 10 days. Clinical responses to decitabine (DAC) were annotated using modified Cheson criteria for AML: RD = refractory disease; Early death = death prior to response assessment; CR = complete remission; less than 5% bone marrow blasts with complete count recovery; CRp = CR without platelet recovery; CRi = CR with incomplete count recovery; HI-P Major = hematologic improvement, platelets. ND = not determined.

The induction of *NY-ESO-1* mRNA was confirmed in a group of 7 German AML patients (Cohort B) receiving decitabine [24]. In this cohort 5/7 sampled patients demonstrated increased expression of *NY-ESO-1* mRNA (Figure 1B). Based upon our initial evaluation of CTA gene induction with HMAs, we elected to further examine induced *NY-ESO-1* and *MAGEA3/A6* expression in a larger cohort of AML patients as these genes are established tumor antigens with clinically translatable vaccines in development.

### Decitabine induces hypomethylation of *LINE-1* elements in serially sampled AML blasts

In order to confirm the results observed in our initial small cohorts of patients, we procured serially sampled peripheral blood samples from a third cohort (C) of 22 HMA naive AML patients receiving decitabine induction at a dose of 20 mg/m^2^/day for 10 consecutive days of a 28 day cycle. Clinical characteristics for the cohort “C” patients are presented in Table 1. As a positive control for changes in global methylation we analyzed *Long Interspersed Nuclear Elements-1* (*LINE-1*) methylation changes using sodium bisulfite pyrosequencing of peripheral blood samples harvested from a range of time periods after the start of decitabine therapy. There was a statistically significant decrease in global methylation when comparing samples obtained pre-decitabine to the nadir *LINE-1* methylation value for each individual patient over time (Supplemental Figure 3). Average *LINE-1* methylation decreased in samples harvested sequentially over the treatment period. A majority of patients show the expected pharmacodynamic response to decitabine treatment.

### Decitabine monotherapy results in hypomethylation of the *NY-ESO-1* and *MAGEA3/A6* promoters and induces gene expression in serially sampled AML blasts

**Figure 2:**
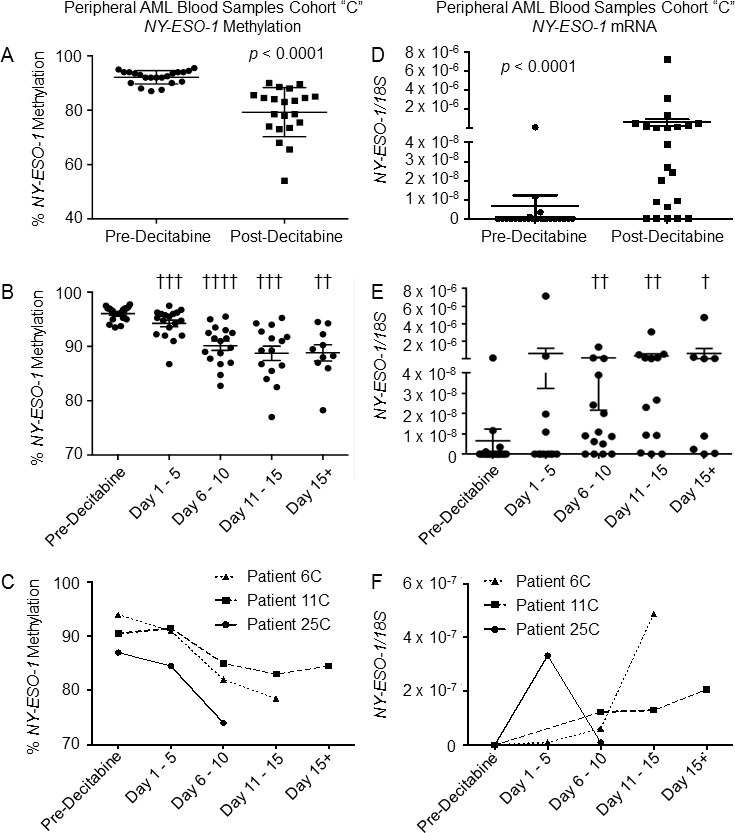
Effect of decitabine monotherapy on *NY-ESO-1* demethylation and mRNA levels in AML peripheral blood cells Analysis of peripheral blood samples harvested from Patient Cohort “C”. **A.** Percentage of methylated *NY-ESO-1* promoters in patient samples harvested pre-decitabine and post-decitabine (*n* = 21). Each “post-decitabine” data point represents the nadir of *NY-ESO-1* methylation across all sampled time points. **B.** Percentage of methylated *NY-ESO-1* promoters in patient samples harvested pre-decitabine compared to samples harvested at days 1 - 5 (*n* = 18), 6 - 10 (*n* = 17), 11 - 15 (*n* = 15) and 15+ (*n* = 10) following day 1 of decitabine therapy. **C.** Percentage of methylated *NY-ESO-1* promoters in serial samples harvested from three patients (6C, 11C, 25C) pre-decitabine and during the first decitabine cycle. Samples for individual patients were binned into 5 day periods. **D.** *NY-ESO-1* mRNA levels in patient samples harvested pre-decitabine and post-decitabine (*n* = 22). mRNA levels were determined using absolute quantification and normalized to *18S* rRNA levels (see Methods). Each “post-decitabine” data point represents the highest *NY-ESO-1* mRNA level for each patient across all sampled time points. **E.** *NY-ESO-1* mRNA levels in patient samples harvested pre-decitabine compared to samples harvested at days 1 - 5 (*n* = 12), 6 - 10 (*n* = 15), 11 - 15 (*n* = 13) and 15+ (*n* = 8) following day 1 of decitabine therapy. **F.** *NY-ESO-1* mRNA levels in serial samples harvested pre-decitabine and during the first decitabine cycle from Patients 6C, 11C, and 25C. Data presented are the average with SEM. For all panels, horizontal bars represent mean values, error bars represent SEM, and p-values were determined using Wilcoxon matched-pairs signed rank test. † = *p* < 0.05; †† = *p* < 0.01; ††† = *p* < 0.001; †††† = *p* < 0.0001

We quantified *NY-ESO-1* and *MAGEA3/A6* promoter methylation and mRNA levels following decitabine monotherapy using our larger cohort “C” of serial patient samples. We determined changes in *NY-ESO-1* promoter methylation using sodium bisulfite pyrosequencing. We first examined the entire cohort, comparing *NY-ESO-1* methylation pre-decitabine to the post-decitabine nadir time point for each patient and observed a statistically significant decrease in *NY-ESO-1* promoter methylation (Figure 2A). Hypomethylation of the *NY-ESO-1* promoter was examined during each of the time intervals as described for *LINE-1* (Figure 2B). As expected, there was a statistically significant decrease in methylation of the *NY-ESO-1* promoter in samples harvested during each post-decitabine period (days 1-5; 6-10; 11-15; 15+). Changes in *NY-ESO-1* and *LINE-1* methylation were significantly correlated (R^2^ = 0.77, *p* < 0.0001, Supplemental Figure 4A). Patients generally demonstrated a progressive decrease in *NY-ESO-1* methylation during treatment. Representative time course data for individual patients is presented for “Cohort C” patients 6, 11 and 25 (Figure 2C).

Prior to decitabine treatment, 18% (4/22) of samples exhibited detectable levels of *NY-ESO-1* mRNA, albeit at very low level (Figure 2D). Following decitabine therapy, 78% (17/22) of samples had detectable levels of *NY-ESO-1* mRNA. Treatment with decitabine was associated with a significant increase in *NY-ESO-1* expression when comparing pre-treatment expression to the maximum expression at any time interval post decitabine. *NY-ESO-1* expression was significantly increased at time points beyond day 6 (Figure 2E). Representative time courses for gene expression are presented for three patients in Figure 2F. Some patients (such as Patient 6) had low to undetectable levels of *NY-ESO-1* at early time points and exhibited a substantial increase in *NY-ESO-1* mRNA during treatment while others (*e.g.* Patient 11) displayed a steady increase in *NY-ESO-1* mRNA throughout the time course. Patient 25 demonstrated early induction of *NY-ESO-1* mRNA which then declined to low/undetectable levels at later time points over the 28 day course. Tight correlation was not observed between *NY-ESO-1* mRNA expression and *NY-ESO-1* promoter methylation across the entire cohort (R^2^ = 0.01, *p* = 0.64, Supplemental Figure 4B).

**Figure 3:**
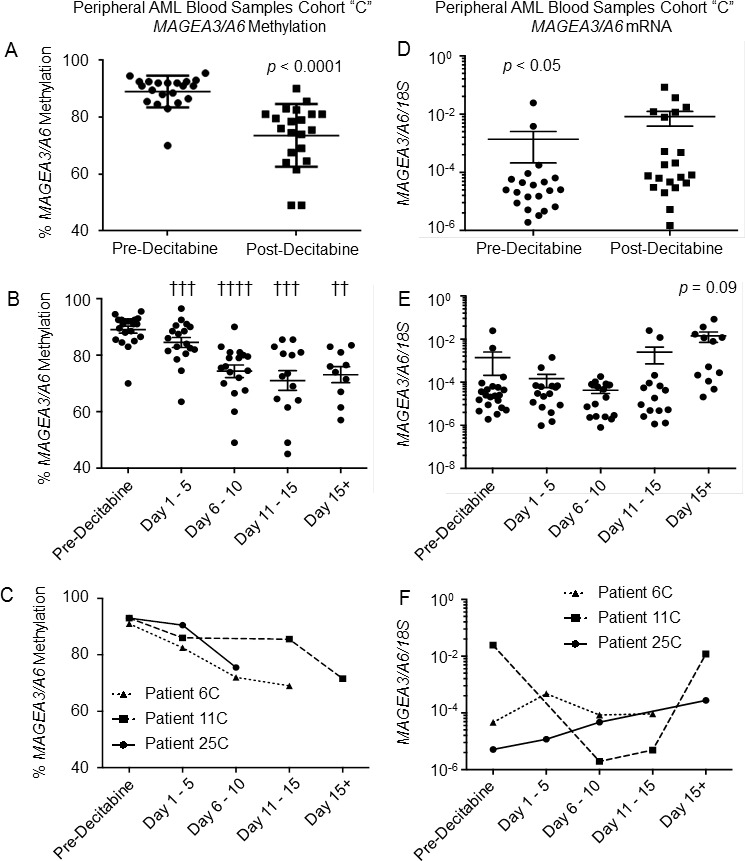
Effect of decitabine monotherapy on *MAGEA3/A6* demethylation and mRNA levels in AML peripheral blood cells **A.** Percentage of methylated *MAGEA3/A6* promoters in patient samples harvested pre-decitabine and post-decitabine (*n* = 21). Each “post-decitabine” data point represents the nadir of *MAGEA3/A6* methylation across all sampled time points. **B.** Percentage of methylated *MAGEA3/A6* promoters in patient samples harvested pre-decitabine compared to samples harvested at days 1 - 5 (*n* = 18), 6 - 10 (*n* = 18), 11 - 15 (*n* = 14) and 15+ (*n* = 10) following day 1 of decitabine therapy. **C.** Percentage of methylated *MAGEA3/A6* CpG residues in serial samples harvested from Patients 6C, 11C, and 25C pre-decitabine and during the first decitabine cycle. Samples were binned into 5 day periods. **D.** Average *MAGEA3/A6* mRNA levels in patient samples harvested pre-decitabine and post-decitabine (*n* = 21). Each “post-decitabine” data point represents the highest *MAGEA3/A6* mRNA level for each patient. mRNA levels were determined using absolute quantification and normalized to *18S* rRNA levels and are plotted on a log_10_ scale. **E.** *MAGEA3/A6* mRNA levels in patient samples harvested pre-decitabine compared to samples harvested at days 1 - 5 (*n* = 16), 6 - 10 (*n* = 16), 11 - 15 (*n* = 15) and 15+ (*n* = 12) following day 1 of decitabine therapy. **F.** *MAGEA3/A6* mRNA levels in serial samples harvested pre-decitabine and during the first decitabine cycle from Patients 6C, 11C, and 25C. For all panels, horizontal bars represent mean values, error bars represent SEM, and p-values were determined using Wilcoxon matched-pairs signed rank test. †† = *p* < 0.01; ††† = *p* < 0.001; †††† = *p* < 0.0001

Compared with *NY-ESO-1*, *MAGEA3/A6* promoter methylation was more heterogeneous in pre-treatment AML samples. Post-decitabine there was a statistically significant decrease in *MAGEA3/A6* promoter methylation when comparing baseline methylation to the post-decitabine nadir time point (Figure 3A). Hypomethylation of the *MAGEA3/A6* promoter was also statistically significantly lower at each time interval studied following decitabine treatment (Figure 3B). As observed for *NY-ESO-1*, methylation of the *MAGEA3/A6* promoter decreased progressively over the sampled time points as exemplified by cohort C patients 6,11 and 25 (Figure 3C). Changes in *MAGEA3/A6* and *LINE-1* methylation were significantly correlated (R^2^=0.4, *p* < 0.01, Supplemental Figure 4A).

Low level *MAGEA3/6* mRNA expression was detected in 100% of diagnostic samples (21/21), and there was a statistically significant increase in *MAGEA3/A6* expression when comparing expression pre-decitabine with the maximal expression any time point following treatment with decitabine (Figure 3D). Induced expression of *MAGEA3/A6* was most increased at the latest time points studied (Days 15+; Figure 3E). Expression of *MAGEA3/A6* also demonstrated some time associated variability, as observed for *NY-ESO-1*, but a majority of patients had later induction of *MAGEA3/A6* as exemplified by patients 6, 11 and 25 (Figure 3F). There was no correlation observed between *MAGEA3/A6* promoter methylation and *MAGEA3/A6* mRNA expression (R^2^ = 0.005, *p* = 0.44, Supplemental Figure 4C).

**Figure 4:**
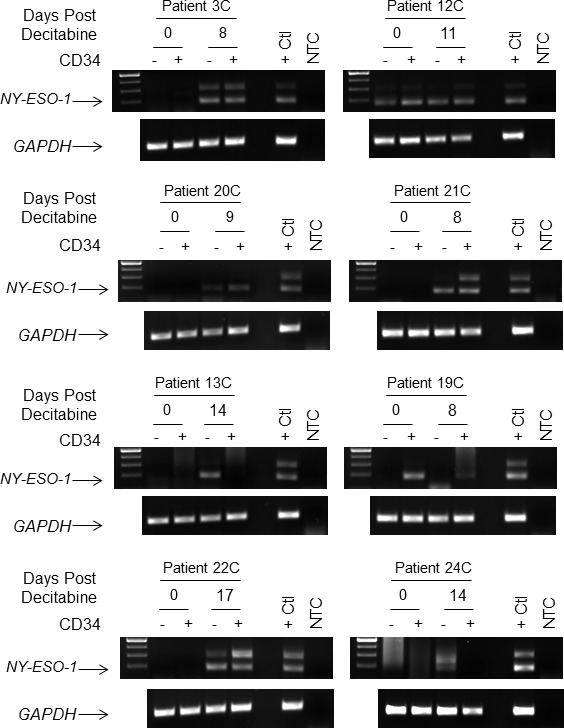
Induction of *NY-ESO-1* in CD34-enriched peripheral blood AML cells following decitabine monotherapy Peripheral blood samples from patients in Cohort “C” (Roswell Park, *n* = 8) were harvested pre- and post-decitabine and CD34 selection was performed. RT-nested PCR was performed to detect the *NY-ESO-1* expression in the CD34 negative (−) and positive (+) fractions. *GAPDH* was used as the loading control. Decitabine-treated OVCAR cells served as the positive control (+ Ctl); no template was used as the negative control (NTC).

To determine whether the observed induction of CTAs was present in the AML blast population, we isolated mRNA from CD34+ and CD34- cells from peripheral blood samples. Samples for this analysis were selected based upon the known blast immunophenotype. For this analysis we focused on *NY-ESO-1* expression due to sample limitations. Nested RT-PCR analysis (required due to low cell numbers, particularly in the post-treatment samples) revealed that 6 out of 8 patients analyzed exhibited *NY-ESO-1* expression in CD34+ blasts following decitabine treatment (Figure 4), indicating induction of *NY-ESO-1* occurred in the AML blasts. *NY-ESO-1* positivity in the CD34+ blast compartment was consistent with *NY-ESO-1* positivity in the unselected bulk mononuclear cell population used for the primary analysis.

### *NY-ESO-1* induction occurs in decitabine-treated AML patients regardless of clinical response

**Figure 5:**
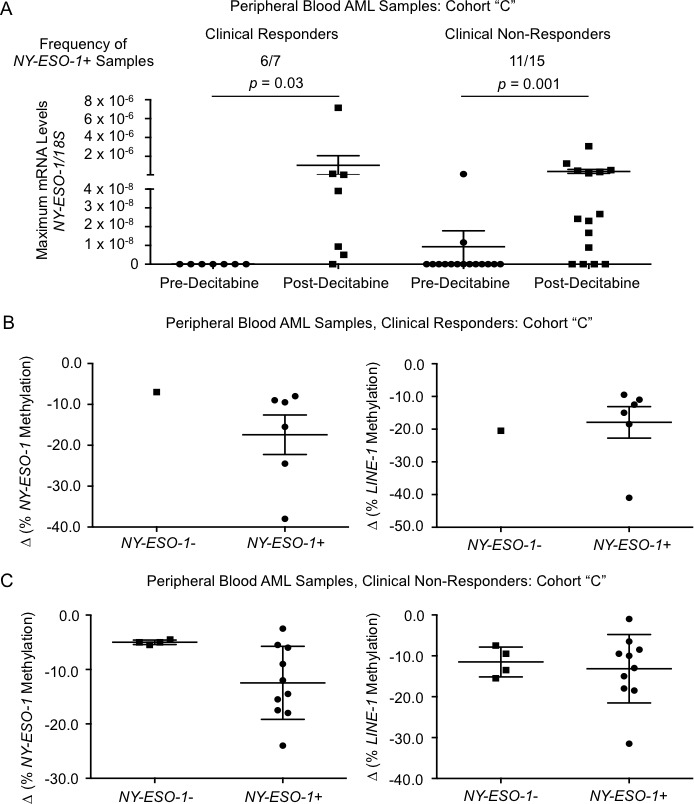
*NY-ESO-1* mRNA levels in AML peripheral blood cells from clinical responders *versus* non-responders to decitabine **A.** Average *NY-ESO-1* mRNA levels in paired samples collected pre-decitabine and post-decitabine (data represent the highpoint of *NY-ESO-1* mRNA levels for each individual patient across multiple time-points and are presented on a log_10_ scale). Patients were separated into clinically responsive (left, *n* = 7) *and* non-responsive (right, n = 15) cohorts based on standard evaluation criteria (see Table 1). Frequencies of samples in each cohort that exhibited detectable levels of *NY-ESO-1* mRNA in the post-decitabine samples are depicted. Absolute levels of *NY-ESO-1* mRNA levels were normalized to *18S* rRNA. **B.** Average post-decitabine change (Δ) of *NY-ESO-1* promoter methylation (left) and *LINE-1* methylation (right) of *NY-ESO-1* mRNA negative (“-“, *n* = 1) and positive samples (“+”, *n* = 6) in clinical responders. **C.** Average post-decitabine change (Δ) of *NY-ESO-1* promoter methylation (left) and *LINE-1* methylation (right) of *NY-ESO-1* mRNA negative (“-“, *n* = 4) and positive samples (“+”, *n* = 10) in clinical non-responders. For **B.** and **C.**, post-decitabine samples were selected based on the methylation nadir across all sample time points. For all panels, horizontal bars represent mean values, error bars represent SEM, and p-values were determined using Wilcoxon matched-pairs signed rank test.

Our analysis of *LINE-1* methylation indicated that the majority of patients responded to decitabine treatment at the molecular level. Hypomethylation has not, however, been tightly correlated with clinical response to decitabine [25–27]. We therefore tested whether levels of *NY-ESO-1* or *MAGEA3/A6* mRNA induction were different in patients who demonstrated a clinical response compared with those who did not. Overall, 7/22 patients (32%) demonstrated a clinical response to decitabine characterized according to the International Working Group (IWG) criteria for AML and MDS (CR, CRp/i or HI) [28, 29]. Sixty-eight percent of patients (15/22) did not demonstrate a clinical response, or died before a response could be evaluated. Of the 7 patients who clinically responded to decitabine, 6 demonstrated a significant increase in *NY-ESO-1* mRNA (Figure 5A). Crucially, *NY-ESO-1* mRNA levels were also significantly increased in 11 out of the 15 patients that did not demonstrate a clinical response to decitabine. Baseline levels of *MAGEA3/A6* were detectable for all the patients studied in Cohort C and there were no statistically significant difference in *MAGE A3/A6* expression following decitabine for responders or non-responders (Supplemental Figure 5A). Changes in *MAGEA3/A6* methylation were also not different between responders and non-responders to decitabine (Supplemental Figure 5B).

We compared *NY-ESO-1* promoter methylation and *LINE-1* methylation with induction of *NY-ESO-1* expression as a categorical variable in both clinical responders and non-responders. Although discrepancies in group sizes preclude us from making a direct comparison using statistical analysis, there appears to be a trend towards lower *NY-ESO-1* promoter methylation in those patients that demonstrated higher levels of *NY-ESO-1* mRNA following decitabine in both clinical responders and non-responders (Figure 5B and 5C). Interestingly, post decitabine *LINE-1* methylation levels were generally lower (mean change −18.3%) among responders than among non-responders (mean change −12.7%), but this was not a statistically significant difference.

### Effect of decitabine treatment on levels of immunoregulatory molecules

In addition to their ability induce the expression of potentially tumor associated antigens like NY-ESO-1 and MAGEA3/A6, HMAs have been shown to have effects on the induction of co-stimulatory and immunoregulatory molecules on cancer cells, potentially enhancing their recognition by the immune system. We and others have demonstrated that *in vitro* exposure to HMAs can increase levels of MHC class I and the costimulatory molecules CD80 and CD86 [18, 30, 31]. Furthermore changes in expression of the immune checkpoint inhibitor PD-L1 in samples exposed to HMAs have been reported [32]. Finally, alterations in antigen processing, such as downregulation of *Transporter Associated with Antigen Processing 1* (*TAP1*) expression, have been reported in a variety of different cancer types and have been shown to limit antigen recognition by the immune system [33]. To test whether decitabine has an effect on expression of any of these immune regulatory molecules, we performed flow cytometry on viably frozen peripheral blood blasts harvested from Cohort C patients between days 4 and 10 after the start of decitabine. In agreement with previous reports, we observed a trend toward increased levels of the MHC Class I molecules HLA-ABC in AML blasts after decitabine treatment compared to the level pre-decitabine (*p* < 0.06; Supplemental Figure 5A) [18]. We also examined expression of the MHC Class II molecule HLA-DR, but did not observe significant changes in its expression (Supplemental Figure 6A). There were likewise no significant changes in the expression of the co-stimulatory molecules CD80 and CD86 following decitabine exposure (Supplemental Figure 6B). It is important to note that due to limitations of sample availability, these data represent a single follow up time point for each patient and are therefore potentially limited by time point selection. In contrast with previous reports, expression of PD-L1 was frequent on AML blasts both before and after treatment with decitabine and did not appear to be substantially changed following exposure to decitabine (Supplemental Figure 6B)[32].

Previous studies demonstrated that the *TAP1* gene, which participates in antigen processing, has a CpG island and is frequently methylated in cancer [34, 35]. Therefore, we tested whether *TAP1* mRNA levels were increased in patients’ samples following decitabine therapy. Individual samples collected from patients undergoing decitabine treatment exhibited a modest increase in *TAP1* mRNA levels compared to their diagnostic sample, but overall, we did not observe a significant increase in *TAP1* mRNA levels (Supplemental Figure 6C).

### AML cells from patients receiving decitabine treatment stimulate cytotoxic NY-ESO-1 specific CD8+ T cells

**Figure 6:**
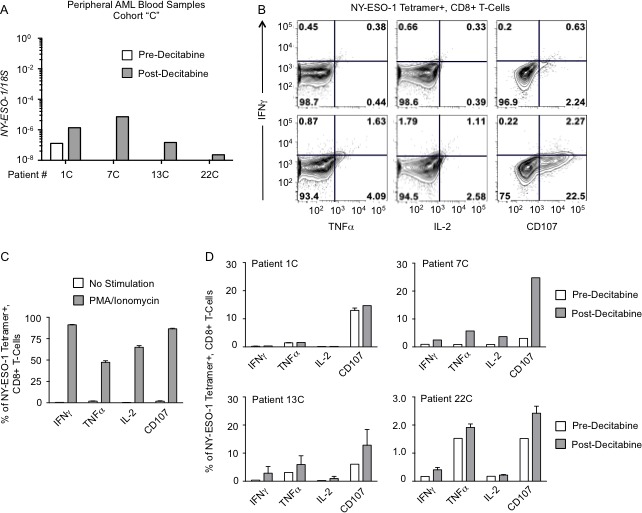
AML peripheral blood cells activate NY-ESO-1-specific T cells following decitabine monotherapy Peripheral blood samples collected from AML patients during the first cycle of decitabine therapy were co-cultured with HLA-*0201 compatible NY-ESO-1 specific CD8+ T-cells. All samples were collected from Cohort “C”. **A.** *NY-ESO-1* mRNA levels for Cohort C patients 1, 7, 13, and 22 pre-decitabine (white bar) and post-decitabine (gray bar). **B.** Representative flow cytometry analysis of T-cell response in HLA-*0201 NY-ESO-1 specific CD8+ T cells following co-culture with peripheral blood cells collected from Patient 7C pre-decitabine (top) and post-decitabine (bottom). NY-ESO-1 specific cells were detected using an NY-ESO-1 specific tetramer. T-cell responses were measured by intracellular cytokine staining for IFNγ (y-axis for all plots), TNFα (left), IL-2 (middle) and expression of cell-surface CD107 (right). **C.** Bar graph depicting percentage of NY-ESO-1 specific CD8+ T cells producing IFNγ, TNFα, and IL-2 and expressing cell-surface CD107 following stimulation with PMA and ionomycin (positive control, gray bar) **D.** Bar graph depicting percentage of NY-ESO-1 specific CD8+ T cells producing IFNγ, TNFα, and IL-2 and expressing cell-surface CD107 following co-culture with HLA-compatible AML peripheral blood cells collected pre-decitabine (white bar) and post-decitabine (gray bar). Error bars depict range of values from 2 technical replicates.

Although we observed a consistent increase in *NY-ESO-1* mRNA levels in the circulating blasts of Cohort C patients treated with decitabine, this low-level mRNA expression does not necessarily translate into a level of protein expression sufficient to trigger recognition and cell killing by antigen specific T cells. We demonstrated detectable NY-ESO-1 protein by immunoblotting on selected patient samples following decitabine therapy, but a majority of patients with detectable *NY-ESO-1* expression did not demonstrate protein expression by western blot (Supplemental Figure 7). We sought to determine whether AML blasts with variable degrees of *NY-ESO-1* mRNA expression were producing sufficient protein expression (below the level of detection by immunoblotting) to allow for recognition by HLA compatible NY-ESO-1 specific CD8+ T-cells. HLA-A*0201 restricted NY-ESO-1-specific CD8+ T cells were obtained from an ovarian cancer patient vaccinated against NY-ESO-1 on a clinical study as previously described [36]. Samples with higher and lower *NY-ESO-1* mRNA expression were used to determine if a threshold of expression was required for T-cell recognition were selected based upon HLA-A*0201 expression by HLA typing. We stimulated NY-ESO-1- specific CD8+ T cells with AML blasts isolated from 4 HLA-A*0201+ AML patients before and after decitabine treatment. The mRNA levels of *NY-ESO-1* in these patients are presented in Figure 6A; 3 of the 4 patients included in this analysis had no detectable NY-ESO-1 protein by immunoblotting. T-cell responses were determined by intracellular cytokine staining for IFNγ, TNFα and IL-2 in NY-ESO-1_157-165_ tetramer-positive CD8+ T-cells. We also quantified the expression of CD107a/b, which is a surrogate marker for the cytotoxic activity of T-lymphocytes. A representative example of the flow cytometry analysis for one patient is shown in Figure 6B. As a positive control for T-cell activation, expression of these markers following non-specific T-cell activation with phorbol 12-myristate 13-acetate (PMA)/ionomycin is shown in Figure 6C. Following co-culture with AML blasts harvested post-decitabine, we observed an increase in the levels of IFN-γ, TNF-α, IL-2, and CD107a/b in HLA-A*0201/NY-ESO-1_157-165_ tetramer+ CD8+ T-cells in three of four patients studied, compared to T-cells co-cultured with AML blasts obtained prior to decitabine exposure (Figure 6B and 6D). These data indicate that peripheral blood blasts harvested from patients receiving standard of care decitabine as induction therapy present sufficient quantities of NY-ESO-1 protein to result in a response from HLA compatible NY-ESO-1-specific CD8+ T cells.

## DISCUSSION

CTA-directed immunotherapy has been employed against tumors that constitutively express CTA genes [15–17]. We and others have demonstrated that several tumor types, including AML, show low to variable transcription of CTA genes due to promoter hypermethylation; exposure to HMAs increases mRNA and protein levels of CTA family members [18–21]. In this report, we have extended our prior findings by demonstrating that AML patients at two separate institutions receiving standard doses and schedules of decitabine exhibit increased mRNA and protein levels of the CTA genes *NY-ESO-1* and *MAGEA3/A6* in circulating blasts. Critically, our data indicate that decitabine treatment results in AML cells that express NY-ESO-1 at levels sufficient for recognition by antigen specific T cells.

We observed variation in both the magnitude and the kinetics of CTA induction in response to decitabine. Although it is possible that is partly due to variation among patients in their individual response to decitabine, our data demonstrating a significant decrease in *LINE-1* methylation suggests that a majority of patients exhibited a pharmacodynamic response to decitabine. There were significant correlations between demethylation of *LINE-1* and demethylation of *NY-ESO-1* and *MAGEA3/A6*, further supporting the interpretation that variance in the mRNA levels of *NY-ESO-1* and *MAGEA3/A6* are not solely due to insufficient demethylation. Although we did not observe a significant correlation between promoter methylation and mRNA level for either *NY-ESO-1* or *MAGEA3/A6,* similar findings have been made in other contexts, suggesting that mechanisms in addition to DNA methylation control expression of these genes [37–39].

There was no apparent association between induction of *NY-ESO-1* and clinical response. The majority of patients who did not respond to decitabine still exhibited an increase *NY-ESO-1* mRNA. The sample size of this cohort does not have sufficient power to determine whether clinical responders exhibit higher or more frequent induction of CTA expression compared to clinical non-responders. Our data indicating that *NY-ESO-1* induction is observed in a majority of clinically non-responsive patients suggest the intriguing possibility that immunotherapies that recognize *NY-ESO-1* have the potential to be effective even in patients who do not respond to decitabine alone.

Our data suggesting an overall trend towards increased levels of HLA Class I molecule expression on AML blasts following decitabine therapy is supported by studies published by our group and others demonstrating that HMAs induce HLA Class I expression in leukemia and other tumor cell lines [18, 31]. The functional significance of this result is still unclear and further work is required to test the hypothesis that HMAs enhance antigen presentation.

Several authors have demonstrated enhanced expression of PD-L1 and a T-cell exhaustion phenotype within the context of disease progression in AML, both in mouse models as well as in primary patient samples [40–43]. Patients with myeloid malignancy treated with HMAs have been shown to have increased expression of checkpoint inhibitory molecules within the malignant cellular compartment, and HMAs may also produce hypomethylation of the PD-1 promoter in circulating lymphocytes [32, 44]. PD-L1 expression was common in our patient samples both before and after treatment with decitabine. Several immunotherapeutic approaches using CTAs, including vaccination and adoptive transfer of T-cells with engineered T-cell receptors or chimeric antigen receptors, have been reported [16, 17, 36, 45–47]. Taken together our data support the hypothesis that combinations of CTA directed vaccination, an HMA and a checkpoint inhibitor might be an attractive approach for patients with AML [32].

## MATERIALS AND METHODS

### Patient samples

Two cohorts of AML patients (“A” and “C”) receiving decitabine monotherapy were enrolled under an Institutional Review Board approved protocol at the Roswell Park Cancer Institute (RPCI). Bone marrow and peripheral blood samples were collected prior to decitabine treatment and peripheral blood samples were collected serially two to four times per week during their first cycle of decitabine therapy (20 mg/m^2^ per day for 10 days). Mononuclear cells were separated and cryopreserved following Ficoll centrifugation. A third cohort of patients (“B”) was treated at the University of Freiberg Medical Center and samples were collected with approval of the Ethics Committee [48]. Clinical characteristics of patients are shown in Table 1 and were previously published in Claus, *et al*. [24].

### Reverse transcriptase quantitative PCR (RT-qPCR)

Absolute quantification of RNA was performed using PCR Master Mix for SYBR Green assays (Eurogentec, Fremont, CA) for *MAGEA3/6* and Taqman probe assay for *NY-ESO-1*-Hs00265824_m1 and *18s rRNA* (cat no-4319413E) (Life Technologies, Carlsbad, CA) on the 7300 Real-Time PCR System (Applied Biosystems, Carlsbad, CA). All samples were run in duplicate, and *NY-ESO-1* and *MAGEA3/6* gene expression data were normalized to *GAPDH* or *18S rRNA*. Primer sequences for *NY-ESO-1, MAGEA3/A6,* and *18S rRNA* have been previously described [18]. Primer sequences for other CTA genes are as follows: *MAGEA1* (Forward: GCACCTCTTGTATCCTGGAGTC; Reverse: GACACTCTCCAGCATTTCTGCC); *MAGEB2* (Forward: GAACCCTGGAAGCTCATCACCA; Reverse: GCTGGTTTCAGCATAGGCTCTC); *PASD1* (Forward: GAAGAGAGGACTTGGTTGCTGC; Reverse: GGAGATCAGGAATGACAACGTGG); *PRAME* (Forward: ACCTGGAAGCTACCCACCTT; Reverse: AGATGCATCACATCCCCTTC); *SP17* (Forward: GGAGTAAGGTAGAAGACCGCTTC; Reverse: TGGTGACTGATGTCTCTTCCTCC); *XAGE1* (Forward: ACCACACAGCCAGTCCCAGGAGCC; Reverse: AACCAGCTTGCGTTGTTTCAGCTTG). GAPDH primers (Forward:TGAAGGTCGGAGTCAACGGA; Reverse:CCATTGATGACAAGCTTCCCG).

Relative quantification of *TAP-1* mRNA levels were determined using the 2^−ΔΔC^_T_ method as previously described [49] and were measured using the Hs_TAP1_QF_1 QuantiFast Probe Assay (Qiagen, Valencia, CA) and normalized to *18S* rRNA.

### Immunoblotting analysis

Whole protein was extracted and quantitated as previously described [50]. 30-100 μg of protein was loaded onto a NuPAGE^®^ Novex^®^ 4-12% Bis-Tris gel (Invitrogen) and transferred to a nitrocellulose membrane (Invitrogen). 5% blotting grade blocker (Bio-Rad, Hercules, CA) in phosphate-buffered saline was used to block nonspecific binding. Membranes were incubated overnight at 4°C with NY-ESO-1 (Invitrogen, clone E978) or MAGE-A antibodies (Invitrogen, clone 6C1) at 1:200, then incubated with secondary antibody (GE Healthcare Life Sciences, Piscataway, NJ) at 1:3000 dilution for 1hr. β-actin antibody (MP Biomedicals, Santa Ana, CA, clone C4) at 1:10,000 dilution was used as a loading control. Proteins were visualized using an enhanced chemiluminescence detection kit (GE Healthcare Life Sciences). As a positive control for NY-ESO-1 expression, we used protein derived from OVCAR-3 cells treated with decitabine as previously described [51].

### Quantitative bisulfite pyrosequencing

The All Prep DNA/RNA Mini kit (Qiagen) was used to isolate genomic DNA and sodium bisulfite conversion was performed using the EZ DNA Methylation Kit (Zymo Research, Irvine CA). Methylation of the *NY-ESO-1* and *MAGEA3/A6* promoters and the *LINE-1* repetitive elements was determined by sodium bisulfite pyrosequencing as previously described [51, 52].

### RT-nested PCR

CD34 positive and negative cells were isolated from peripheral mononuclear cells using CD34 Microbeads as per manufacturer instructions (Miltenyi Biotec). RNA and cDNA was prepared as described earlier [18]. *NY-ESO-1* nested PCR was performed by performing two PCR reactions: PCR-1 (Forward: 5′-CAGGGCTGAATGGATGCTGCAGA-3′ and Reverse: 5′-GCGCCTCTGCCCTGAGGGAGG-3′; amplifying a 332 bp product) followed by PCR-2 (Forward: 5′-GGCTGAATGGATGCTGCAGA-3′ and Reverse: 5′-CGGACACAGTGAACTCCTTC-3′; amplifying 177 bp product) [18, 53]. *GAPDH* primers sequences are as mentioned earlier. PCR products were amplified with initial denaturation at 95°C for 5 min, then 35 cycles of denaturation at 95°C for 30 sec., annealing at 60°C for 30 sec., and extension at 72°C for 30 sec., followed by a final 5 min extension at 72°C. PCR products were analyzed on 2% agarose gel by ethidium bromide staining.

### Flow cytometry

Cells were stained with mouse anti-human CD34 (Allophycocyanin (APC)-conjugated, clone 4H11; eBioscience, San Diego, CA), HLA-DR (Brilliant Violet (BV) 711-conjugated, clone L243; BioLegend, San Diego, CA), HLA-A,B,C (APC-Cy7-conjugated, clone W6/32; BioLegend), PD-L1 (Phycoerythrin (PE) -conjugated, clone 29E.2A3; BioLegend), CD80 (BV650-conjugated, clone 2D10; BioLegend), and CD86 (BV605-conjugated, clone IT2.2; BioLegend). Live cells were determined by staining cells with 4′,6-diamidino-2-phenylindole (DAPI) and were defined as DAPI-negative. Cells were analyzed using an LSRII (Becton Dickinson, Franklin Lakes, NJ) and raw data were analyzed using FlowJo v.9.5.2 software (TreeStar, Ashland, OR).

### NY-ESO-1 specific CD8+ T cell recognition assay

NY-ESO-1-specific HLA-A*0201-restricted CD8+ T cells were co-cultured with HLA-A*0201 bone marrow or peripheral blood mononuclear cells pre and post-decitabine treatment from AML patients for 6 hr at 37°C in the presence of anti-CD107a (clone H4A3) and CD107b (clone H4B4) [36]. Monensin and brefeldin A were added during the last 4 hr of incubation to block cytokine secretion. Cells were fixed with 2% formaldehyde, followed by permeabilization staining with IFNγ (clone B27), TNFα (clone MAb11) and IL-2 (clone MQ1-17H12) in the presence of normal mouse IgG and permeabilization buffer (Invitrogen-Caltag). Negative and positive control stimulations with and without peptide (NY-ESO-1_157-165_) or PMA and ionomycin were set up in parallel.

### Statistical analysis

All statistical analyses were performed using GraphPad Prism 6. For all experiments, p-values were determined using non-parametric Wilcoxon signed rank tests or Spearman rank correlations, *p* values < 0.05 were deemed significant.

## SUPPLEMENTARY MATERIAL FIGURES


