# Supplementary material for: Induction of cancer testis antigen expression in circulating acute myeloid leukemia blasts following hypomethylating agent monotherapy
Source: Oncotarget. 2016 Feb 11;7(11):12840–56. doi: 10.18632/oncotarget.7326 (PMC4914325; doi:10.18632/oncotarget.7326)
Supplement: Supplementary file 1 [file oncotarget-07-12840-s001.pdf]

Induction of cancer testis antigen expression in circulating acute myeloid leukemia blasts following hypomethylating agent monotherapy

Supplementary Material

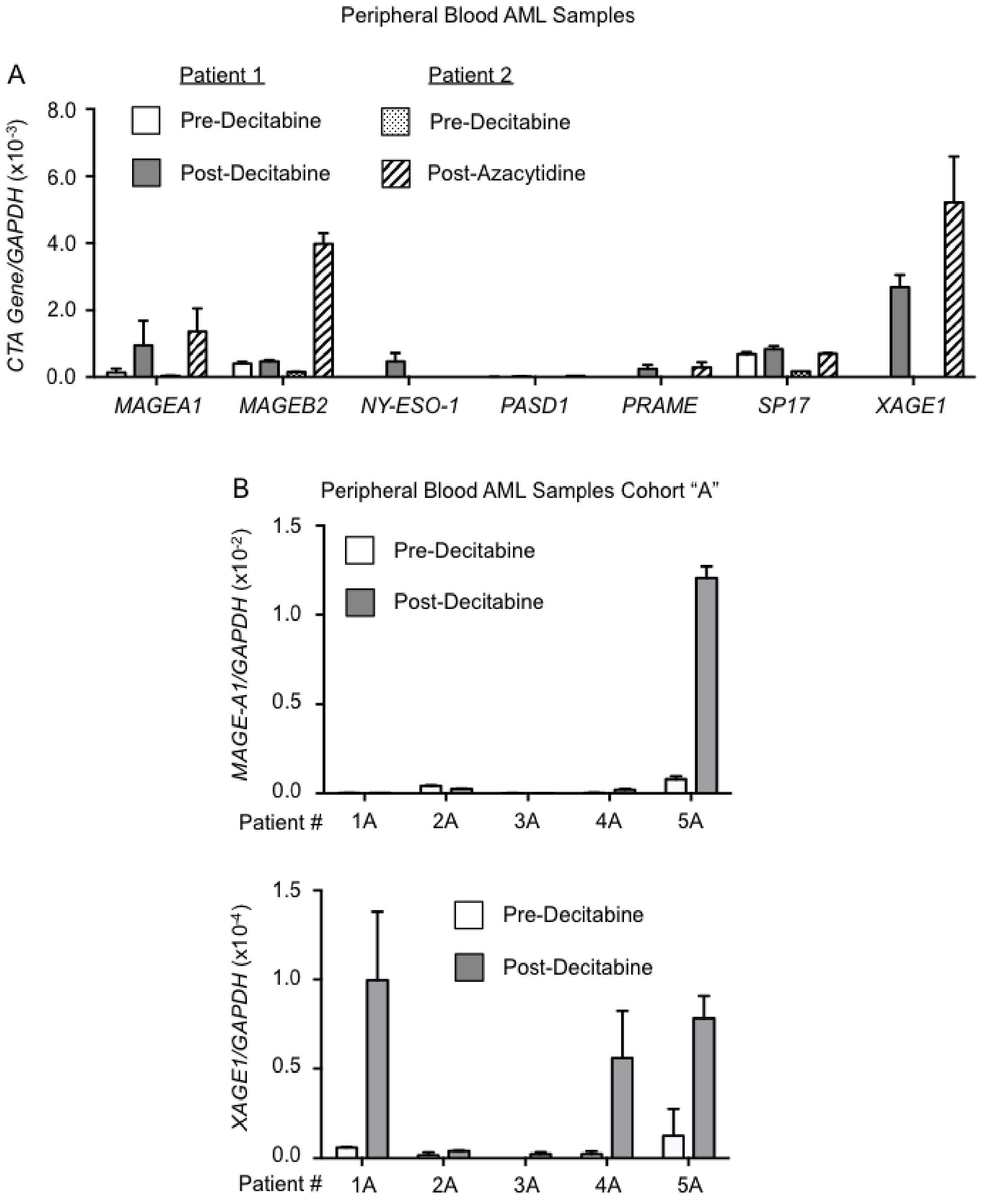

Supplemental Figure 1. mRNA levels of cancer testis antigen genes in AML peripheral blood cells following treatment with hypomethylating (HMA) agents. (A) Quantitative PCR analysis of mRNA levels of various cancer testis antigen (CTA) genes was performed on AML peripheral blood cells harvested from one patient (Patient 1) who received decitabine monotherapy (pre-decitabine = white bar; post-decitabine = gray bar) and another patient (Patient 2) who received azacitidine monotherapy (pre-decitabine = spotted bar; post-azacitidine = hatched bar). Samples were obtained during the first cycle of HMA therapy and represent the highest CTA mRNA level for each patient. (B) Quantitative PCR analysis of *MAGEA1* (top) and *XAGE1* (bottom) mRNA levels obtained pre-decitabine (white bar) and post-decitabine (gray bar) from Cohort “A” (n = 5, Table 1 for clinical characteristics). For all panels, mRNA levels were determined using absolute quantification and normalized to *GAPDH* mRNA levels. Error bars depict SEM of 2 technical replicates for each patient sample.

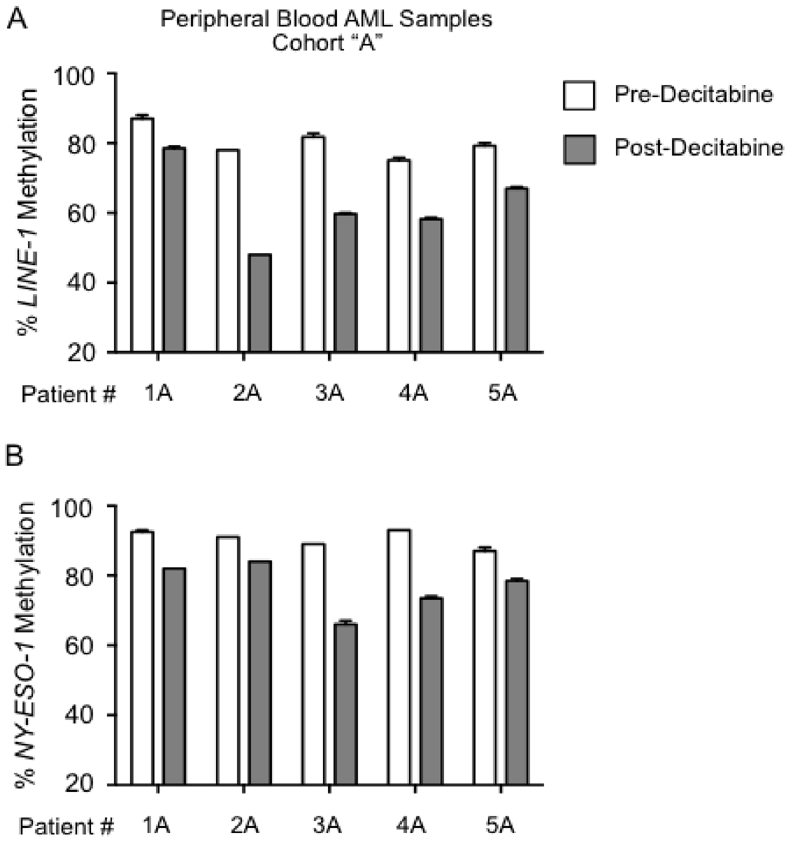

Supplemental Figure 2. Demethylation in AML peripheral blood cells following decitabine monotherapy. Analysis of DNA methylation of peripheral blood samples harvested from Patient Cohort "A". (A) Percentage of methylated *LINE-1* promoters in patient samples harvested pre-decitabine and post-decitabine (n = 5). *LINE-1* methylation was used as a surrogate for global methylation and was determined using pyrosequencing. Each "post-decitabine" data point represents the nadir of *LINE-1* methylation across all sampled time points. (B) Percentage of methylated *NY-ESO-1* promoters in patient samples harvested pre-decitabine and post-decitabine (n = 5). Each "post-decitabine" data point represents the nadir of *NY-ESO-1* methylation across all sampled time points. Error bars depict SEM of 2 technical replicates for each patient sample.

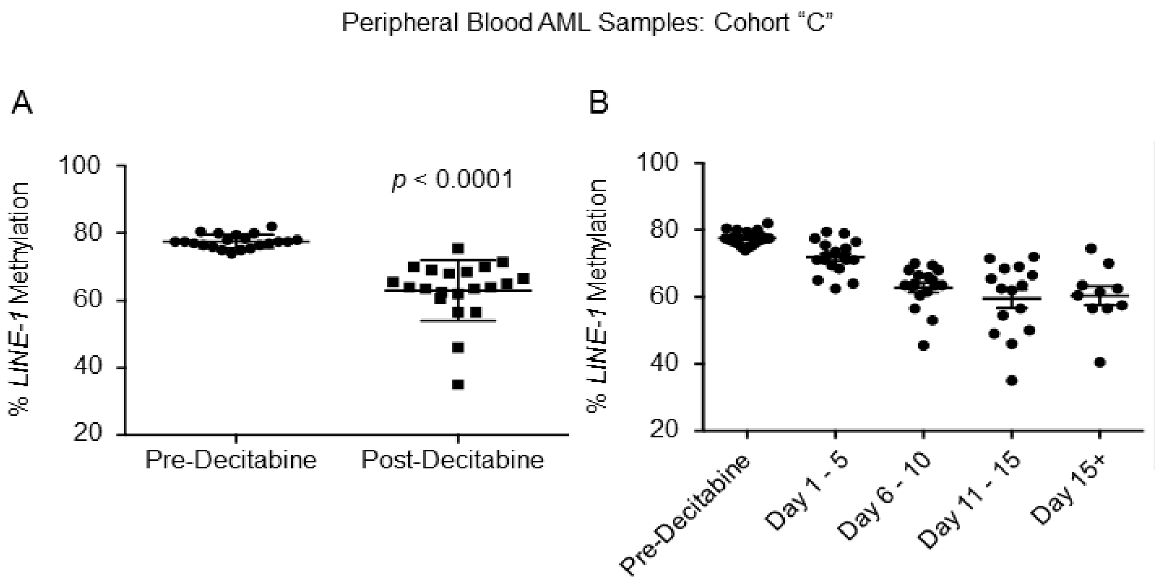

Supplemental Figure 3. Global demethylation in AML peripheral blood cells following decitabine monotherapy. Peripheral blood samples from Patient Cohort "C" (Roswell Park) (see Table 1) were serially harvested pre-decitabine and at serial points following administration of decitabine. *LINE-1* methylation was used as a surrogate for global methylation and was determined using pyrosequencing (A). Percentage of methylated *LINE-1* elements in patient samples harvested pre-decitabine and post-decitabine (n = 21). Each "post-decitabine" data point represents the nadir of *LINE-1* methylation across all sampled time points. The horizontal bar represents the mean value and error bars represent the SEM. (B). *LINE-1* methylation changes across the cohort during the sampled time course. Percentage of methylated *LINE-1* elements in patient samples harvested pre-decitabine compared to samples harvested at days 1 – 5 (n = 18), 6 – 10 (n = 17), 11 – 15 (n = 15) and 15+ (n = 10) following day 1 of decitabine therapy. p-values were determined using Wilcoxon matched-pairs signed rank test.

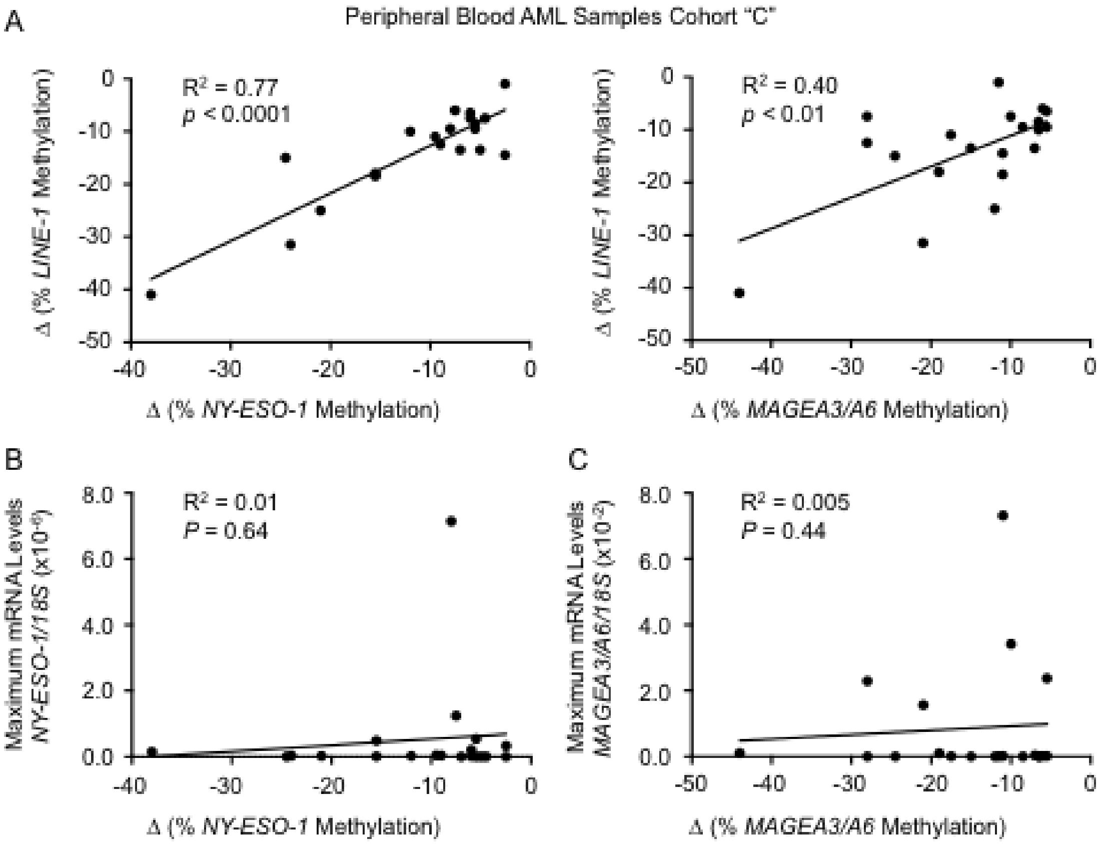

Supplemental Figure 4. Association of *NY-ESO-1* and *MAGEA3/A6* promoter methylation with mRNA levels. (A). Correlation between changes ("Δ") in *LINE-1* methylation and *NY-ESO-1* promoter methylation (left, n = 20) and *MAGEA3/A6* promoter methylation (right, n = 20) in AML peripheral blood samples harvested from Cohort "C" patients post-decitabine. (B) Correlation between maximum mRNA levels and change in promoter methylation for *NY-ESO-1* (left, n = 20) and *MAGEA3/A6* (right, n = 20) in AML peripheral blood samples harvested from Cohort "C" patients post-decitabine. For all panels, R^2 values were determined by linear regression and p-values were determined by Spearman rank correlation.

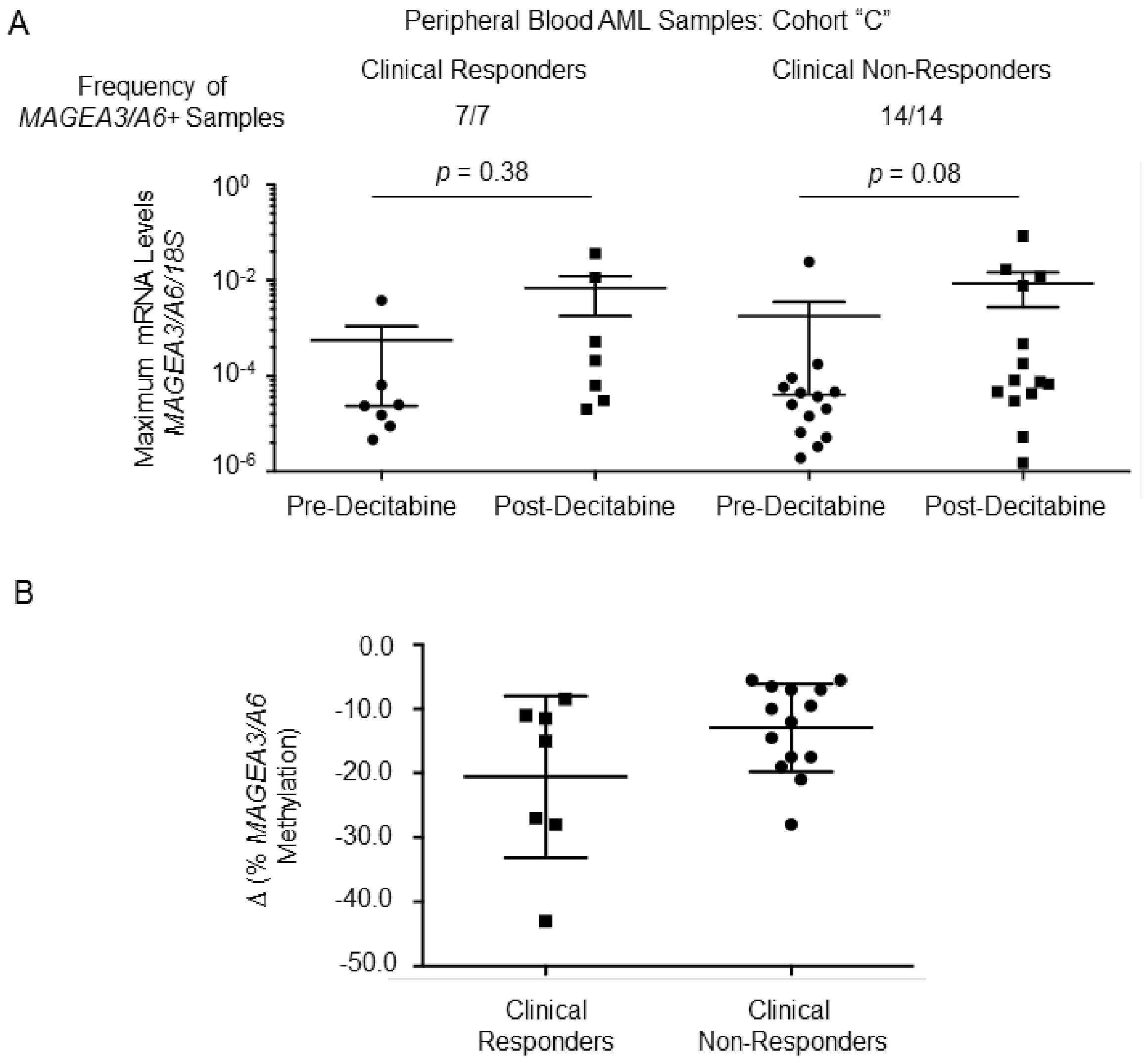

Supplemental Figure 5. *MAGEA3/A6* mRNA levels in AML peripheral blood cells from clinical responders *versus* non-responders to decitabine. (A). Average *MAGEA3/A6* mRNA levels in paired samples collected pre-decitabine and post-decitabine (data represent the highpoint of *MAGEA3/A6* mRNA levels for each individual patient across multiple time-points). Patients were separated into clinically responsive (left, n = 7) *and* non-responsive (right, n = 14) cohorts based on standard evaluation criteria (see Table 1). Frequencies of samples in each cohort that exhibited detectable levels of *MAGEA3/A6* mRNA in the post-decitabine samples are depicted. Absolute levels of *MAGEA3/A6* mRNA levels were normalized to *18S* rRNA and are

plotted on a $\log_{10}$ scale. (B). Average post-decitabine change (Δ) of *MAGEA3/A6* promoter methylation in clinical responders and non-responders. Post-decitabine samples were selected based on the methylation nadir across all sample time points. For all panels, horizontal bars represent mean values, error bars represent SEM, and p-values were determined using Wilcoxon matched-pairs signed rank test.

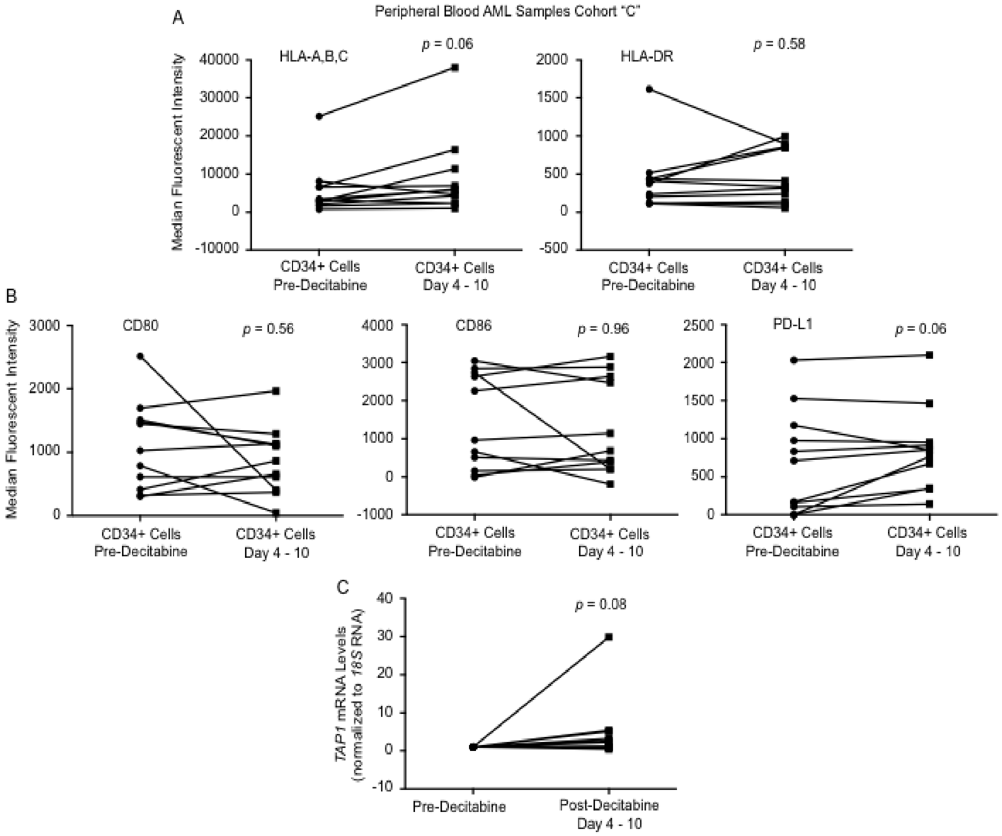

Supplemental Figure 6. Effect of decitabine monotherapy on levels of immunoregulatory molecules in AML peripheral blood cells. (A) Median fluorescent intensity of HLA-A,B,C (left) and HLA-DR (right) in CD34+ cells from paired patient samples harvested pre-decitabine and between 4 to 10 days following start of decitabine treatment (n = 11). (B) Median fluorescent intensity of CD80 (left), CD86 (middle), and PD-L1 (right). Paired samples are indicated by connecting lines. CD34, HLA-ABC, HLA-DR, CD80, CD86, and PD-L1 levels were determined using flow cytometry. (C). *TAP1* mRNA levels in paired patient samples harvested pre-decitabine and between 4 to 10 days following start of decitabine treatment. *TAP1* levels were determined using relative quantification and normalized to *18S* rRNA. For all panels, p-values were determined using Wilcoxon matched-pairs signed rank test.

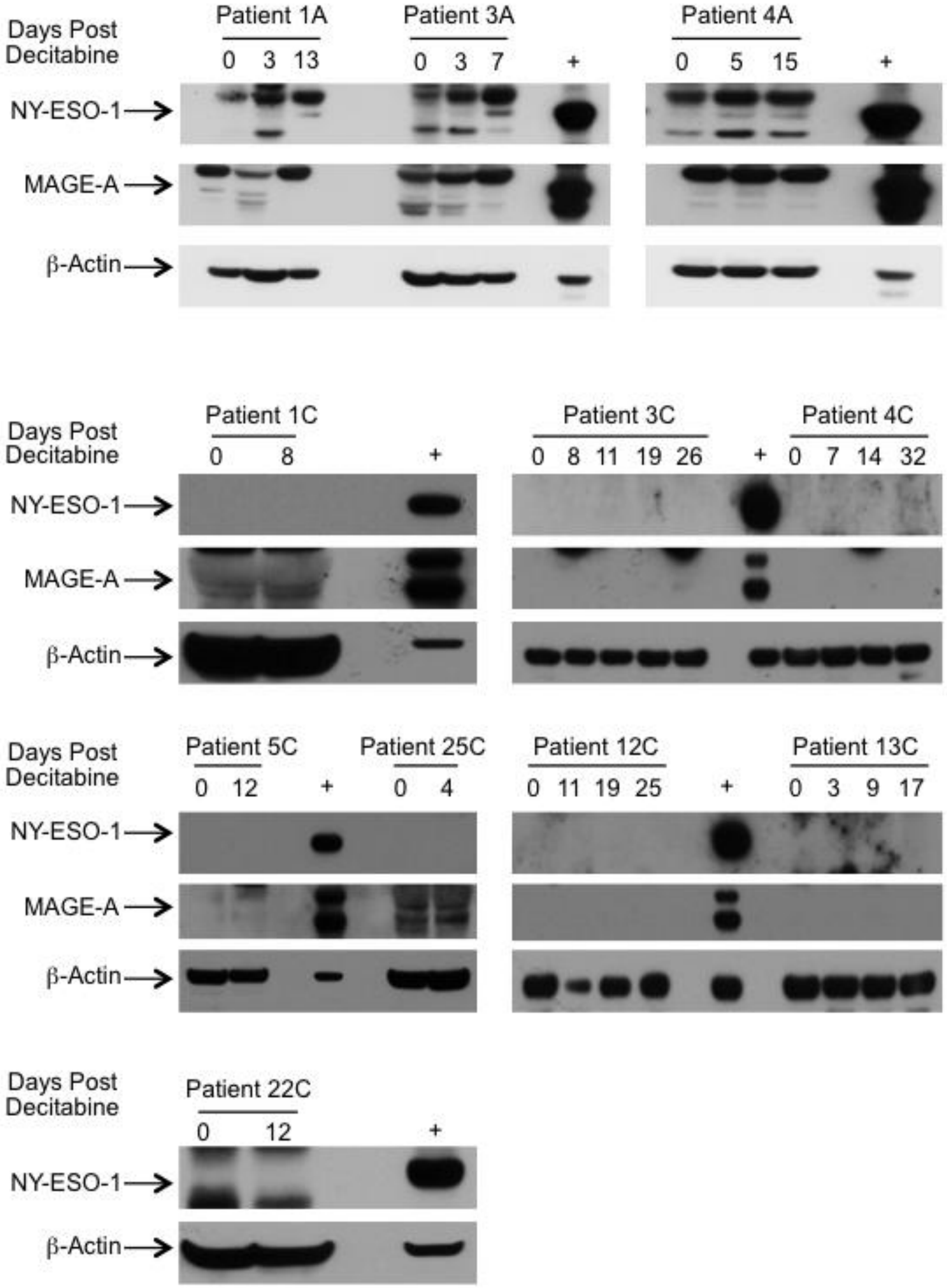

Supplemental Figure 7. Effect of decitabine monotherapy on NY-ESO-1 and MAGEA protein levels. Immunoblotting analysis of NY-ESO-1 and MAGEA protein levels in serial peripheral blood samples harvested from 3 Cohort “A” patients and 8 Cohort “C” patients. β-actin was used as a loading control and OVCAR cells exposed to decitabine acted as a positive control (“+”).
